# Curcumin: A Potent Protectant against Esophageal and Gastric Disorders

**DOI:** 10.3390/ijms20061477

**Published:** 2019-03-24

**Authors:** Slawomir Kwiecien, Marcin Magierowski, Jolanta Majka, Agata Ptak-Belowska, Dagmara Wojcik, Zbigniew Sliwowski, Katarzyna Magierowska, Tomasz Brzozowski

**Affiliations:** Department of Physiology, Faculty of Medicine, Jagiellonian University Medical College, 16 Grzegorzecka Street, 31-531 Cracow, Poland; skwiecien@cm-uj.krakow.pl (S.K.); m.magierowski@uj.edu.pl (M.M.); jolmaj@poczta.fm (J.M.); agata.ptak-belowska@uj.edu.pl (A.P.-B.); dagmara1.wojcik@uj.edu.pl (D.W.); AgaZS@poczta.fm (Z.S.); katarzyna.magierowska@uj.edu.pl (K.M.)

**Keywords:** curcumin, reflux esophagitis, gastroprotection, gastric ulcer, *Helicobacter pylori*, gastric cancer

## Abstract

Turmeric obtained from the rhizomes of Curcuma longa has been used in the prevention and treatment of many diseases since the ancient times. Curcumin is the principal polyphenol isolated from turmeric, which exhibits anti-inflammatory, antioxidant, antiapoptotic, antitumor, and antimetastatic activities. The existing evidence indicates that curcumin can exert a wide range of beneficial pleiotropic properties in the gastrointestinal tract, such as protection against reflux esophagitis, Barrett’s esophagus, and gastric mucosal damage induced by nonsteroidal anti-inflammatory drugs (NSAIDs) and necrotizing agents. The role of curcumin as an adjuvant in the treatment of a *Helicobacter pylori* infection in experimental animals and humans has recently been proposed. The evidence that this turmeric derivative inhibits the invasion and proliferation of gastric cancer cells is encouraging and warrants further experimental and clinical studies with newer formulations to support the inclusion of curcumin in cancer therapy regimens. This review was designed to analyze the existing data from in vitro and in vivo animal and human studies in order to highlight the mechanisms of therapeutic efficacy of curcumin in the protection and ulcer healing of the upper gastrointestinal tract, with a major focus on addressing the protection of the esophagus and stomach by this emerging compound.

## 1. Introduction

Curcumin, the natural phenolic active ingredient of turmeric (Curcuma longa) rhizome, has been used in Asia as an herbal remedy for a variety of diseases [1]. Similar to chili, turmeric is commonly used in Asian cuisine to add a yellow color, both as a flavor and as a preservative [2]. In addition to the use of curcumin as an anti-inflammatory in ancient times, it has also been used to treat gastrointestinal (GI) diseases such as indigestion, flatulence, diarrhea, and even gastric and duodenal ulcers [1,2,3]. Recently, great attention has been paid to the medical applications of curcumin in the treatment of human diseases associated with oxidative stress and inflammation, including different cancers [3]. Curcumin treatment has also led to the improvement of metabolic parameters involving aging-associated diseases such as atherosclerosis, diabetes, cardiovascular disease, and chronic kidney diseases [4,5]. Interestingly, some promising effects of curcumin have been observed in the alleviation by this turmeric derivative of the chronic inflammatory conditions such as arthritis, uveitis, and inflammatory bowel disease [6]. In some instances, curcumin has been found to aid in the prevention and treatment of various cancers [7]. Recently, the anticarcinogenic activity of curcumin has been documented in the GI tract because this compound has proven to exert a therapeutic effect on different human GI cancers such as esophageal, gastric, and small and large intestinal cancer [8,9]. This overview was aimed to document the beneficial and emerging effects of curcumin in the upper GI tract, focusing on the mechanism of local, systemic, and molecular actions of this compound in the esophagus and stomach.

## 2. Curcumin in the Protection of the Esophagus against Reflux Esophagitis, Barrett’s Esophagus, and Esophageal Carcinoma

The esophagus, which carries food and liquid from the mouth to the stomach, undergoes transient lower esophageal sphincter relaxation (TLESR), which is considered the main mechanism of gastroesophageal reflux disease (GERD) [10]. Under physiological conditions, these TLESRs are induced spontaneously without swallowing and allow for the “physiological” contact of gastric juice containing hydrochloric acid (HCl) with the esophageal wall [10]. Interestingly, this acid reflux occurs at a higher frequency during TLESR in patients with GERD than in healthy subjects [10,11]. Furthermore, the anatomical abnormalities in the structure of the lower esophageal sphincter or its dysfunction can result in more frequent or sometimes prolonged exposure of the esophageal mucosa to gastric acid, resulting in esophageal damage due to reflux esophagitis. If this mucosal contact of epithelial cells with acid or acid and bile (mixed reflux) is prolonged, GERD develops [12]. Thus, human esophageal epithelial cells are a direct target and play a key role in esophageal inflammation in response to acidic pH in the course of GERD development. Complications of GERD development include Barrett’s esophagus with an increased risk for esophageal adenocarcinoma formation [13]. In a study designed to mimic the acid exposure experienced by GERD patients, treatment with curcumin prevented the expression of inflammatory cytokines in human esophageal tissue [14]. This anti-inflammatory effect of curcumin has been confirmed by an in vitro study, testing the protective potential of curcumin in esophageal epithelial cell lines exposed to exogenous acid [15]. That study examined the HET-1A cell lines exposed to hydrochloric acid in relation to the role of PKC, MAPK, and NFκB signaling pathways and the transcriptional regulation of IL-6 and IL-8 expression [15]. This HET-1A cell line appears to be suitable for investigating the cellular action of putative esophageal metaplasia development, and both acid and bile are considered potent carcinogenic factors in the mechanism of esophageal dysplasia and adenocarcinoma formation [15,16,17]. The exposure of HET-1A cells to pH 4.5 induced the activity of the transcription factor NF-κB while enhancing IL-6 and IL-8 secretion and their mRNA and protein expression [15]. Among the kinases system tested in their study [17], particularly the MAPKs and PKC (alpha and epsilon) activity were activated when HET-1A cells were exposed to acid. Curcumin was equally potent as SN-50 (an NF-κB inhibitor), chelerythrine (a PKC inhibitor), and PD-098059 (a p44/42 MAPK inhibitor), yet all of them efficiently abolished the acid-induced mucosal expression of IL-6 and IL-8 [17]. 

In in vivo studies, curcumin was compared with lansoprazole, the proton pump inhibitor (PPI) commonly used as the recommended standard drug against GI-tract disorders including GERD [18,19]. In these reports, curcumin was shown to effectively prevent the esophageal mucosal damage induced by acute reflux esophagitis [18,19]. Although curcumin was documented as less potent than the proton pump inhibitor (PPI) lansoprazole in the inhibition of acid reflux esophagitis, it became superior to lansoprazole in the inhibition of mixed acid-bile reflux-induced esophagitis. This protective mechanism caused by curcumin in the esophagus has been attributed to the antioxidant nature of this turmeric derivative [18,19]. 

Gastroesophageal reflux is a major mechanism responsible for Barrett’s metaplasia, which develops from the cellular reprogramming of the esophageal squamous epithelium due to the reflux of acidic or acidic-bile content to the esophagus [20]. The protection against oxidative stress and the preservation of the antioxidative activity induced by esophageal protectants play an important role in the strategy against the pathogenesis of acid-induced esophageal mucosa damage. Thus, the anti-reflux therapy alternative to PPIs is widely expected. In line with this notion, the anti-inflammatory properties of curcumin and the relationship between bile-reflux and the expression of antioxidative enzymes and the reactive oxygen metabolites (ROM) scavenging enzyme MnSOD have been investigated [21]. The molecular approach was to examine therapies effective in the preservation of both the expression of the antioxidative enzyme MnSOD at the level of the protein and the enzymatic activity of MnSOD [21]. Curcumin applied in the form of oil exerted an esophagoprotective activity against acidic reflux injury through its ability to maintain mitochondrial function, as documented by the preservation of both the MnSOD expression and activity by this turmeric compound [21]. These authors concluded that curcumin oil prevented the loss of MnSOD expression in the rat esophageal epithelium caused by bile [21]. In addition, the treatment with curcumin oil prior to acid or bile salt exposure prevented the loss of MnSOD activity in an esophageal HET-1A cell line. Notably, when cells were treated with curcumin oil, the highest level of MnSOD enzyme activity was observed [19]. However, it is worth to mention that besides curcumin, MnTBAP and other nutraceuticals including certain berry extracts also offered the efficient preservation of MnSOD expression in HET-1A cells [21]. 

**Figure 1:**
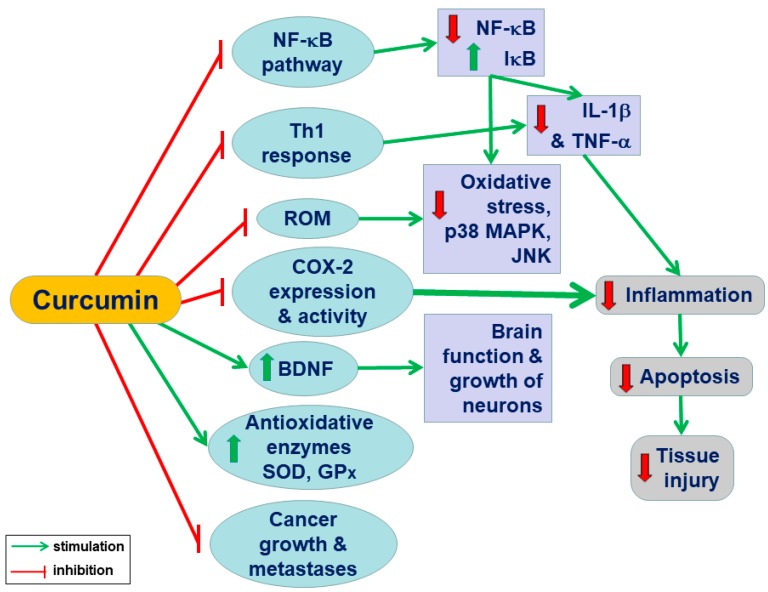
A conclusive summary of the pleiotropic action of curcumin in the body system: Curcumin exhibits anti-inflammatory, antioxidant, antiapoptotic, antitumor, and antimetastatic activities and suppresses multiple signalling pathways responsible for inflammation, apoptosis, and cellular death. Curcumin improves the growth of neurons and the functions of the brain in addition to the downregulation of reactive oxygen species, oxidative stress, and proinflammatory factors (NF-κB and cytokines).

Curcumin may offer a benefit over the single pathway-targeted anticancer therapy, and this effect could be due to its pleiotropic properties [20]. Figure 1 summarizes the pleiotropic effects of curcumin resulting in the amelioration of inflammation and cell death, which protects the tissue against injury. 

Considerable evidence indicates that curcumin can efficiently prevent the acid- and bile-induced NF-κB changes from the normal mRNA phenotype into the oncogenic phenotype of human hypopharyngeal primary cells (HHPC) in culture [22]. Curcumin inhibited the bile acid-induced genes dependent on the NF-κB signalling pathway in this HHPC culture. This turmeric was capable in selectively inhibiting Bcl-2 overexpression induced in HHPC exposed to bile at both acidic (pH 4.0) and neutral (pH 7.0) environments [22]. This evidence clearly indicates that curcumin might be superior over NF-κB inhibitors. Furthermore, its beneficial anti-inflammatory and anticarcinogenic effects are independent of pH status and can be explained either by the promotion of cell apoptosis or the inhibition of antiapoptotic pathway in these HHPC cells [22]. In another study, the efficacy of curcumin in the prevention of bile acid-induced DNA damage using micronucleus assay was investigated [23]. Moreover, these authors tested the effect of curcumin on NF-κB and NF-κB p65 activities in the curcumin-pretreated esophageal cell line (OE33) exposed to deoxycholic acid (DCA) using real-time PCR of the extracted RNA. In their study, the bile-induced DNA damage and the activation of NF-κB activity in vitro were completely abolished by curcumin [23]. An important part of this translational study has been run in human subjects and dealt with the curcumin efficacy to treat patients with Barrett’s esophagus [23]. These patients with Barrett’s esophagus took a daily dose of 500 mg of curcumin tablets for 7 days prior to endoscopy [23]. Interestingly, curcumin-supplemented patients presented a slightly reduced expression of IL-8 as compared to the squamous control tissue non-treated with curcumin. However, this treatment resulted in an almost doubled apoptotic frequency compared to the non-supplemented control patients [23]. To reiterate, the data has clearly indicated that curcumin exerted a beneficial effect against bile-driven deleterious effects on the mucosal esophageal cells. Although curcumin was poorly delivered to the esophagus, the supplementation of patients with Barrett’s esophagus with curcumin not only reduced NF-κB activity and inflammatory features in esophagi but also increased apoptosis in the Barrett’s esophageal tissues. This confirms that apoptosis could be one of the potentially important mechanisms of the curcumin-beneficial effect on squamous esophageal mucosa.

## 3. Curcumin-Induced Gastric Protection against NSAID-Induced Gastric Damage: Experimental and Clinical Evidence

It is well-known that the ingestion of NSAIDs is associated with the risk of GI adverse effects including gastric micro bleeding, damage to the epithelial structure, cessation of GI blood flow, decline in gastric mucus and alkaline secretion, and alteration in GI motility [24]. The mechanism of NSAID-induced gastropathy involves an impairment of the gastric mucosal barrier mainly due to the inhibition of endogenous prostaglandins (PGs), which are considered prototypes of the cytoprotective agents with the ability to protect the gastric mucosa against a variety of topical and non-topical ulcerogenes [25,26]. The therapy with NSAIDs which includes aspirin, the most popular and widely used world-wide, and other drugs, such as indomethacin, diclofenac or naproxen, have especially brought great attention towards their anti-inflammatory potential and efficacy to treat rheumatoid arthritis [24,25,26,27]. The most common adverse effects of NSAIDs documented in experimental animals and confirmed in humans include potent ulcerogenic activity and the enhancement of oxidative stress [27,28,29]. Therefore, the reduction of oxidative stress may be an effective curative strategy for preventing and treating NSAIDs-induced gastric mucosal complications, such as micro bleedings and hemorrhagic lesions. The alternative therapy by phytochemicals such as supplementation by the dietary phenolic compounds including curcumin, could exert antioxidant, anti-inflammatory, and antibacterial benefits, thus preventing digestive diseases of upper GI tract including the ulcerogenic activity of NSAIDs [30]. 

In rodents, the NSAID-induced gastric mucosal damage is mainly localized to the oxyntic mucosa of the stomach’s corpus region. These lesions are recognized as bleeding erosions or lesions rather than typical ulcers [28]. This is contrary to the situation in humans, where NSAID-induced gastric ulceration occurs mainly in the gastric antrum [31]. For instance, naproxen is the most common and frequent NSAID used in rheumatoid arthritis patients, and the naproxen-induced gastropathy occurs mainly in the gastric antrum [31]. Using this experimental antral model of gastric ulcerations in rats to mimic the human scenario of complication risk after naproxen ingestion, Kim et al. [32] have shown that the administration of naproxen caused the macro- and microscopic antral lesions and increased the tissue lipid peroxidation levels. When curcumin was combined with naproxen, the size of the gastric antral ulcers had diminished, followed by the restoration of the activity of antioxidative enzymes SOD, catalase, and glutathione peroxidase (GPx), which are all recognized as ROM scavengers in the gastric mucosa [32]. Notably, curcumin protection was accompanied by the fall in the lipid peroxides, suggesting that the mechanism of curcumin-induced attenuation of gastric mucosal injury caused by NSAIDs such as naproxen can involve the inhibition of lipid peroxidation and activation of radical scavenging enzymes [32]. Thus, the data clearly indicates that curcumin possesses protective anti-antral ulcer properties due to its capability to decrease the damage of antral gastric mucosa. Therefore, the future clinical utility of curcumin may offer an attractive strategy and an encouraging opportunity for curing gastric lesions induced by NSAIDs in humans.

Recent studies confirmed that curcumin is an effective antioxidant and anti-inflammatory compound in the upper GI-tract and a scavenger of ROM and nitrogen metabolites [33]. There is considerable evidence that curcumin, which is not associated with significant adverse effects, exhibits a comparable anti-inflammatory efficacy with those presented by some derivatives of NSAIDs [33,34,35]. Nowadays, it seems obvious that the mechanism of curcumin-induced gastroprotection against indomethacin injury depends on the curcumin-mediated downregulation of pro-inflammatory mediator expression, the decline in free nitrogen radical generation in addition to the enhanced resistance of mucosal epithelium due to the inhibition of apoptosis, and the increased cell proliferation in the gastric mucosa [33]. Ganguly et al. [34] have shown that curcumin is similar to melatonin with regards to its down regulatory action against the activity of metalloproteinase-2 (MMP-2) generated by ROM in animal models of gastric damage and during the course of ulcer healing. In addition, the suppression of MMP-2 activity by H_2_O_2_ in a dose- and time-dependent manner in vitro was blocked by antioxidants including curcumin [34]. Curcumin, similarly to melatonin and omeprazole, afforded gastroprotection in vivo against indomethacin-induced gastric damage by the suppression of gastric mucosal biosynthesis and expression of MMP-2 [34]. Interestingly, they have proposed that the protection of curcumin, melatonin, and the PPI omeprazole against H_2_O_2_-mediated inactivation depends on the downregulation of MMP-2 and TIMP-2 expression and the upregulation of MT1-MMP during the onset of indomethacin-induced ulceration [34]. 

Morsy et al. [35] have confirmed the protective efficacy of curcumin against the damaging activity of indomethacin in the rat stomach. Indomethacin administered intraperitoneally at a dose of 30 mg/kg produced gastric bleeding erosions, predominantly due to the profound inhibition of endogenous PG in the gastric mucosa evoked by this agent [24,25]. The pretreatment with a single dose of curcumin reduced the index of indomethacin-induced gastric lesions and the malondialdehyde (MDA) concentration, which is considered the index of ROM-induced lipid peroxidation [35,36]. Along with the attenuation of the indomethacin-induced gastric mucosal damage, the concomitant increase in mucin content of gastric juice and the gastric mucosal nitric oxide (NO) levels in rats pretreated with curcumin have been observed [36]. Furthermore, the treatment with curcumin raised the antioxidant enzyme catalase and SOD activities and decreased the expression of pro-inflammatory stimuli such as inducible nitric oxide synthase (iNOS) and NF-κB. Curcumin attenuated gastric acid secretory activity, an important component implicated in the pathogenesis of gastrointestinal peptic ulcer disease, and inhibited the activity of caspase-3. This finding suggests that the mechanism of this protection depends on the strengthening of the mucosal barrier and the antioxidant and antiapoptotic activities of this compound. Of note, this beneficial gastroprotective effect of curcumin against NSAIDs could be attributed, at least in part, to the antisecretory activity of this turmeric compound [37]. Interestingly, the addition of microelements such as zinc (Zn) to curcumin enhanced the gastroprotective and ulcer healing activities compared with those exhibited by curcumin alone [37]. Such a complex of Zn(II)-curcumin dose-dependently reduced the severity of the indomethacin-induced gastric damage, as reflected by the lower gastric ulcer index in animals treated with this combination [37]. Using rats with chronic preexisting gastric ulcers, Mei and coworkers concluded that the Zn(II)-curcumin complex efficiently enhanced the mucosal barrier defence activity by the attenuation of oxidative stress and MMP-9-mediated inflammation to a greater extent than curcumin alone [38]. Indeed, the treatment with curcumin raised SOD activity and GSH levels and markedly inhibited the MDA content and the expression of MMP-9 in the ulcerated mucosa [38]. In another report, curcumin-mediated gastric mucosal healing has been associated with the upregulation of genes for MMP-2, TGF-β, and VEGF. These effects were considered essential for an angiogenic modulatory role of this turmeric as documented by its stimulatory effect on the vascular sprout formation and collagen fiber restoration in ulcerated tissues [39]. In nineteen-hour animal ulcer models, Tuorkey and Karolin [40] have shown that the mechanism of anti-ulcer activity of curcumin depends upon the attenuating effect of this compound on gastric acid hypersecretion, total peroxides, MPO activity, IL-6 levels, and apoptotic incidence. This observation was in keeping with the earlier study by Mathattanadul et al., who have also indicated that both curcumin and bisdemethoxycurcumin can inhibit the basal gastric acid secretion in pylorus-ligated rat model and that this antisecretory effect can contribute to an acceleration of the healing of chronic gastric ulcerations of the mucosa [19]. 

To date, only a few studies mentioned above have been conducted—in vitro and especially in vivo—on the inhibitory properties of curcumin affecting gastric secretion. Kim et al. demonstrated that the *Curcuma longa* extract protected the gastric mucosa against ulceration with an extent similar to ranitidine and inhibited gastric acid secretion in rats with pylorus ligation procedure, thus preventing gastric mucosa from gastric ulcerations [41]. Zinc(II)-curcumin complex A also shared similar antisecretory properties because this combination of zinc and curcumin provided protection against indomethacin injury, in part, by the inhibition of gastric acid secretion [42].

The question remains whether the bioavailability of NSAIDs is affected by curcumin or if turmeric shows the genuine gastroprotective action in the stomach. Zazueta-Beltran et al. have demonstrated that the concurrent administration of indomethacin and curcumin resulted in a significant reduction of gastric damage when compared to indomethacin alone [43]. However, the bioavailability parameters of indomethacin and the prodrug acemetacin co-administered with curcumin was not significantly altered after the administration of either the active compound or the prodrug. This important evidence indicates that curcumin exhibits a protective effect against indomethacin-induced gastric damage without marked change in bioavailability or through the pharmacokinetics of NSAIDs such as indomethacin [43].

## 4. Role of Curcumin in the Protection against Gastric Mucosal Injury Induced by Strong Necrotizing Agents and Stress-Induced Gastric Mucosal Bleeding Erosions 

Despite the proven multi-target, anti-inflammatory properties of curcumin, the potential protective action of this turmeric derivative against the gastric mucosal damage induced by noxious agents has not been extensively studied. As a consequence, the mediating factors and mechanisms of the potential protective effects of curcumin in the stomach injured by necrotizing agents such as ethanol are poorly understood. Despite ethanol being known as a strong gastric-damaging agent causing mucosal injury due to its direct contact with the gastric mucosa, ethanol-induced gastropathy constitutes a serious clinical entity in humans [44]. In the original report, Mei et al. [37] have demonstrated that the oral administration of a complex of zinc and curcumin (zinc(II)-curcumin) dose-dependently reduced the severity of ethanol-induced gastric lesions while suppressing the gastric acid secretory activity as reflected by the H^+^/K^+^-ATPase activity comparable with that exhibited by the PPI, lansoprazole. Furthermore, Zn(II)-curcumin significantly inhibited TNF-α and IL-6 mRNA expression, increased the activity of SOD and GPx, and reduced MDA levels in gastric mucosa of rats when compared to the respective controls. These findings suggest that the gastroprotective activity of the Zn(II)-curcumin complex might be important for stimulating cell proliferation and adjusting the pro-inflammatory cytokine-mediated oxidative damage caused by ethanol insult of the gastric mucosa [37]. 

**Figure 2:**
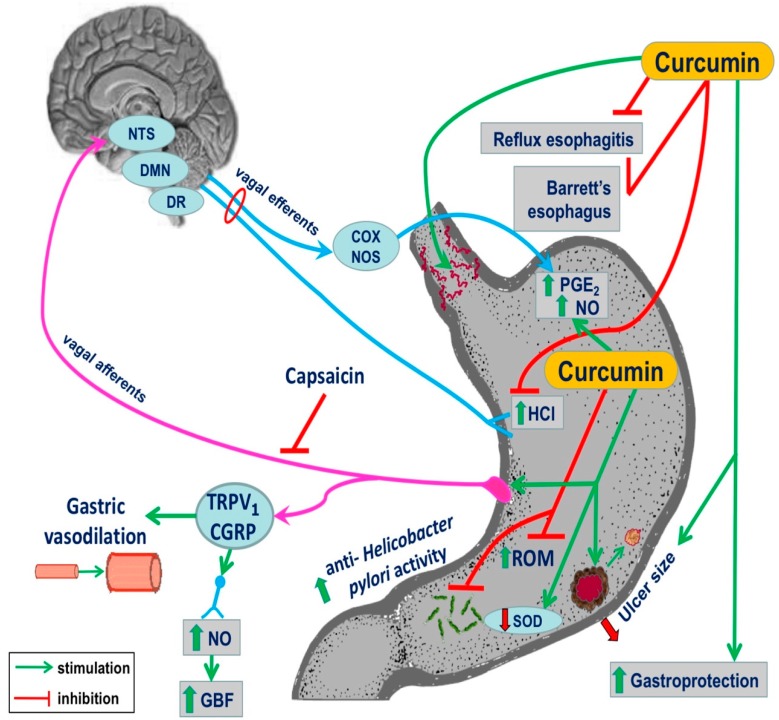
The complex summary of the beneficial effect of treatment with curcumin in esophageal and gastric protection: It involves the amelioration of damage induced by reflux esophagitis and incidence of Barrett’s esophagus, attenuation of inflammation, prevention of gastric damage formation, the anti-*Helicobacter pylori* activity, and an improvement of communication between gut function and the brain (gut–brain axis) by this turmeric derivative to facilitate local microvascular vasodilation and an increase in organ blood flow, gastroprotection, and ulcer healing.

Previous studies have demonstrated that endogenous NO and other gaseous molecules such as H_2_S and CO can cooperate with PG and sensory nerve neuropeptides such as calcitonin gene-related peptide (CGRP) in the mechanism of gastric mucosal integrity and gastroprotection [45,46]. The recent study by Czekaj et al. revealed that some of these factors, such as PG and NO, may contribute to the mechanism of curcumin-induced gastric protection against ethanol injury [47]. They have demonstrated that curcumin given intragastrically provided a dose-dependent gastroprotection against gastric lesions induced by ethanol while increasing both the GBF and the plasma gastrin levels [47]. Furthermore, they proposed that curcumin-induced protection may depend upon the reduced mRNA expression of pro-inflammatory mediators HIF-1α and caudal type home box 2 (Cdx-2), both also recognized as tumour markers in the gastric mucosa [47]. The evidence that curcumin enhanced the gastric mucosal expression of antioxidative enzymes HO-1 and SOD2 indicated that the mechanism of gastroprotection-induced by this turmeric compound involves the enhancement of the antioxidative status of gastric mucosa challenged by ethanol [47]. Interestingly, the mechanism of curcumin-induced protection against ethanol injury could also depend upon the endogenous bioavailability of PG because nonselective and selective COX-1 and COX-2 inhibitors (indomethacin, rofecoxib, and SC-560) reversed this protection and the accompanying rise in GBF evoked by this polyphenolic compound. Furthermore, the concurrent treatment with the synthetic analogue of PGE_2_ combined with these COX-1 and COX-2 inhibitors restored the protective and hyperaemic activities of curcumin against ethanol damage [47]. This clearly indicates that PG can be an important downstream effector of curcumin-evoked beneficial protective action in the stomach. NO, which is an important endogenous mediator of gastroprotection and ulcer healing, could be involved in the mechanisms underlying the gastroprotective activity of curcumin because the L-NNA-induced depletion of NO biosynthesis in the gastric mucosa abolished the gastroprotective effects of curcumin. Moreover, this gastroprotective effect was accompanied by a marked reduction in the GBF. The concurrent treatment with L-arginine together with L-NNA not only restored the protective and hyperaemic activities of curcumin against ethanol injury but also abrogated an increase in the mRNA expression of HIF-1α and Cdx-2 induced by L-NNA. Nowadays it seems likely that CGRP released from sensory afferents as well as vanilloid receptor TRPV1 can cooperate with PG and NO in the mechanism of the gastroprotective action of curcumin against ethanol injury [48,49,50,51]. This notion is supported by the observation that the protection and gastric hyperaemic response induced by curcumin were lost in animals with deactivated sensory nerves by capsaicin and were further restored when exogenous CGRP was concurrently administered with curcumin in rats with capsaicin-deactivated sensory nerves compromised by ethanol (Figure 2) [47].

Recent evidence indicates that curcumin can be effective as a protective substance against the formation of stress-induced gastric lesions in rats [52], in part due to the inhibition of gastric secretory activity mediated by a strong inhibitory action on H^+^/K ^+^ATPase activity in parietal cells of the rat stomach. Using a chromatin immunoprecipitation assay, He et al. have demonstrated that curcumin inhibited the H^+^/K^+^ATPase promoter via histone acetylation, the gene and protein expression of the gastric H^+^/K-ATPase α subunit, thus resulting in the rise in pH and the amelioration of stress-induced gastric ulcerogenesis [52]. In another study, the anti-ulcer activity of curcumin in rats exposed to either chronic stress and/or unpredictable stressors was observed simultaneously with an evident improvement in memory deficit assessed in their study by the elevated plus maze test and by the overall improvement of homeostatic functions [53]. The pretreatment of stressed rats with curcumin via the oral route attenuated chronic stress and chronic unpredictable stress-associated memory deficits and counteracted the increase in TBARS generation and the decrease in GSH content and markers of oxidative stress (corticosterone, glucose, and creatine kinase) [53]. These authors concluded that the curcumin-mediated antioxidant actions help the body to regulate corticosterone secretion and the stress-induced ulcerative action and possibly to play an important central and peripheral adaptive role against chronic and unpredictable stressors [53]. 

The protective activity of curcumin against stress ulcerogenesis can involve the cooperation of endogenous prostaglandins and NO and the activity of capsaicin-sensitive afferent fibers releasing CGRP and capsaicin receptors TRPV1 [54]. Recent studies revealed that the intragastric administration of curcumin dose-dependently prevented the formation of gastric bleeding erosions in gastric mucosa compromised by cold stress. Furthermore, it inhibited basal and histamine- and pentagastrin-stimulated gastric acid secretion, recognized as one of the major pathogenic factors in stress ulcerogenesis [54,55]. Interestingly, the gastroprotective effects of curcumin raised the plasma concentration of gastrin in rats exposed to cold stress [55]. Gastrin is a hormone secreted by the G cells of the APUD cells of the antral gastric mucosa known to exhibit a trophic effect on mucosa, resulting in increasing cell proliferation and gastroprotection against the damaging effects of ethanol and aspirin [56]. Exposure to stress increased the gastric mucosal expression of mRNA for pro-inflammatory markers TNF-α, COX-2, and iNOS, but these effects were inhibited by curcumin administered in graded dosages [55]. The functional ablation of sensory afferent nerves with capsaicin or pretreatment with capsazepine blunted the curcumin-induced decrease in the lesion index and the increase in GBF evoked by cold stress [55]. This vasoactive activity of curcumin corroborates the recent observation that the turmeric derivative, as well as other curcuminoids, can exert vasodilatory activity even in isolated organs [57]. These effects of curcumin in rats with capsaicin denervation were restored by the concomitant treatment with exogenous CGRP combined with curcumin in rats subsequently exposed to cold stress, therefore supporting the role of sensory afferent vasodilatory neuropeptides in gastroprotection by this turmeric derivative against stress-induced gastric damage [55].

## 5. Efficacy of Curcumin to Treat the Impairment the Gastric Mucosa Infected by *Helicobacter pylori* (*H. pylori*)

According to the World Health Organization (WHO), *H. pylori* has been accepted as a first-class human pathogen implicated in the pathogenesis of major disorders of the upper GI-tract such as the development of gastritis, peptic ulcers, MALT lymphoma, and in some cases, gastric adenocarcinoma. The problem of an infection with *H. pylori* is nowadays important in the general population since epidemiological studies have shown that over 50% of the populations is infected with H. pylori, with a much higher rate in developing countries. Therefore, various drugs including antibiotics have been routinely used for the eradication of this infection. However, steadily increasing resistance to antibiotics, undesirable side effects, the raising costs, and the impaired *H. pylori*-infected patient’s quality of life have given rise to the recent surge of interest in alternative approaches [58]. 

Recent evidence revealed that treatment with curcumin can attenuate oxidative stress and the histological changes accompanying chronic gastritis associated with *H. pylori*. In a randomized clinical trial, patients were divided into two groups: a standard triple therapy group and a group treated with triple therapy with the concurrent administration of curcumin [58]. Endoscopic and histological examinations were performed for all patients before and after 8 weeks of treatments [58]. Triple therapy with curcumin adjuvant as a treatment group significantly decreased the MDA markers and glutathione peroxides and increased the total antioxidant capacity of the gastric mucosa at the end of study, compared to the baseline and triple regimen groups without curcumin [58]. In addition, the oxidative damage to DNA was significantly decreased in triple therapy with the curcumin group at the end of study, compared to the baseline and the triple therapy [58]. This important study has documented that curcumin added to the triple therapy markedly attenuated the inflammation scores (active, chronic, and endoscopic) of patients, compared to the baseline and triple therapy group without combination with curcumin [58]. These authors have concluded that curcumin added to the triple anti-*H. pylori* therapy considerably increased the eradication rate, which was superior as compared to triple therapy alone [58]. Using an experimental mouse model, the effects of curcumin on lipid peroxidation level, MPO and urease activity, number of colonized bacteria, levels of anti-*H. pylori* antibodies, biofilm formation, IFN-γ, IL-4, and gastrin and somatostatin levels in serum have been studied [59]. While all parameters were increased in *H. pylori*-infected mice, the treatment with curcumin notably reduced the number of bacteria colonizing gastric mucosa and attenuated the activity of lipid peroxide, MPO and urease strongly supporting the hypothesis that curcumin can reduce the effects of *H. pylori* infection due to its potent antioxidizing properties. Curcumin also exhibited a potent antimicrobial activity against *H. pylori* isolates in mice infected with this bacterium [59]. In contrast, the level of anti-IgG antibodies and somatostatin was increased following curcumin treatment, suggesting that this compound possessed immunomodulation properties resulting in the normalization of the feedback inhibition of gastrin by somatostatin disturbed in *H. pylori*-infected gastric mucosa [59]. Both pro-inflammatory NF-κB and the motogenic response in *H. pylori*-infected epithelial cells were inhibited by curcumin [60]. In recent trials, the addition of curcumin to triple therapy regimes ameliorated the oxidative stress and histopathologic changes in chronic gastritis-associated *H. pylori* infections [61,62,63]. All together, these studies suggest that curcumin can be a useful supplement to improve the gastric mucosal protection against chronic inflammation and may prevent the carcinogenic changes in patients with chronic gastritis associated with *H. pylori* (Figure 2).

## 6. Conclusions and Future Perspectives

The poor water solubility, dissolution, and retention time of curcumin in the stomach limits its practical usefulness in the treatment of peptic ulcer disease and neoplastic alterations including oral, esophageal, and gastric cancers in humans [62,63,64]. However, the therapeutic effect of curcumin might be exerted by its metabolites. For instance, Jamil et al. [57] have studied the spasmolytic, inotropic, and chronotropic activity of major curcumin metabolite tetrahydrocurcumin and the nonenzymatic curcumin hydrolysis products ferulic acid, feruloyl methane, and vanillin. They concluded that demethoxycurcumin and bisdemethoxycurcumin showed more pronounced spasmolytic effects in guinea pig ileum as well as vasodilation and negative inotropic activity in guinea pig arteries and atria, respectively, than those exhibited by a parent curcumin. This evidence seems to indicate that both curcuminoids derivatives can contribute to the observed pharmacological effects of the *C. longa* extract [57]. Thus, future studies are required to prove if the enrichment of extracts of *C. longa* with curcumin metabolites demethoxycurcumin and bisdemethoxycurcumin could potently enhance the therapeutic efficacy of curcumin. Recently, Chen et al. (65) have shown that plasma curcumin was below the detection limit of 0.1 ng/ml after oral curcumin administration in healthy volunteers; instead, only the curcumin metabolite, curcumin glucuronide, has been detected as early as 30 min after curcumin administration and achieved a maximal concentration within 2.7 hours. This suggests a rapid metabolism of curcumin which form the glucuronide conjugate (65). More importantly Chen et al. (65) have revealed that the gene expression of antioxidative genes NRF2, HO-1, and NQO1 was increased and the epigenetic genes for histone deacetylases HDAC1, HADAC2, HADAC3, and HADFAC4 have been suppressed by curcumin glucuronide. They concluded that despite the absence of the parent curcumin in the blood/plasma, the antioxidant and epigenetic modulatory effects of curcumin glucuronide can explain the potential overall health beneficial effect of this herbal medicinal product [65]. Thus, it is reasonable to believe that most of the curcumin effects in vivo may be due to local and direct effects rather than systemic effects of this turmeric compound after absorption. This notion which is supported by the pharmacokinetics and pharmacodynamics of curcumin regulating antioxidant and epigenetic gene expression in humans could be of interest for basic researchers and clinicians. 

However, a recent study with a controlled release therapy of curcumin to treat gastric ulcers by novel raft forming systems incorporating curcumin-Eudragit^®^ EPO solid dispersions has triggered attention with a great hope to develop a curcumin carrier with improved solubility and the dissolution of this compound and its prolonged gastric residence time [66]. Importantly, these authors have demonstrated a curative effect of this curcumin raft on the acetic acid-induced chronic gastric ulcer in rats. The curcumin raft forming formulations at the dose of 40 mg/kg administered once daily showed a superior effect in terms of the acceleration of the ulcer healing, as compared with the standard antisecretory therapy with the PPI lansoprazole (1 mg/kg, twice daily) and a curcumin suspension (40 mg/kg, twice daily) [66]. These studies have indicated that this new raft-forming system containing curcumin solid dispersions could serve as a promising carrier for the specific delivery of poorly soluble lipophilic compounds, such as curcumin, to treat upper GI tract disorders in humans.
